# Role of PI3K in the bone resorption of apical periodontitis

**DOI:** 10.1186/s12903-022-02364-2

**Published:** 2022-08-11

**Authors:** LiNa Wang, Ming Dong, DongMei Shi, CaiHui Yang, Shuo Liu, Lu Gao, WeiDong Niu

**Affiliations:** 1 Department of Endodontics and Periodontics, College of Stomatology, Dalian Medical University, 9 West Section, Lvshun South Road, Dalian, 116044 Liaoning Province China; 2 Department of Pediatric Stomatology, The Third People’s Hospital of Puyang City, Puyang, Henan Province China

**Keywords:** Lipopolysaccharide, Inflammation, Akt, NF-κB

## Abstract

**Background:**

Phosphoinositide 3-kinase (PI3K) is located within cells, and is involved in regulating cell survival, proliferation, apoptosis and angiogenesis. The purpose of this study was to investigate the role of PI3K in the process of bone destruction in apical periodontitis, and provide reference data for the treatment of this disease.

**Methods:**

The relative mRNA expression of PI3K, Acp5 and NFATc1 in the normal human periodontal ligament and in chronic apical periodontitis were analyzed by real-time quantitative polymerase chain reaction (RT-qPCR). A mouse model of apical periodontitis was established by root canal exposure to the oral cavity, and HE staining was used to observe the progress of apical periodontitis. Immunohistochemical staining was used to detect the expression of PI3K and AKT in different stages of apical periodontitis, while enzymatic histochemical staining was used for detection of osteoclasts. An *Escherichia coli* lipopolysaccharide (LPS)-mediated inflammatory environment was also established at the osteoclast and osteoblast level, and osteoclasts or osteoblasts were treated with the PI3K inhibitor LY294002 to examine the role of PI3K in bone resorption.

**Results:**

The expression of PI3K, Acp5 and NFATc1 genes in chronic apical periodontitis sample groups was significantly increased relative to healthy periodontal ligament tissue (*P* < 0.05). Mouse apical periodontitis was successfully established and bone resorption peaked between 2 and 3 weeks (*P* < 0.05). The expression of PI3K and Akt increased with the progression of inflammation, and reached a peak at 14 days (*P* < 0.05). The gene and protein expression of PI3K, TRAP and NFATc1 in osteoclasts were significantly increased (*P* < 0.05) in the *E. coli* LPS-mediated inflammatory microenvironment compared to the normal control group. Meanwhile in osteoblasts, the gene and protein expression of PI3K, BMP-2 and Runx2 were significantly reduced (*P* < 0.05) in the inflammatory microenvironment. With the addition of LY294002, expressions of bone resorption-related factors (TRAP, NFATc1) and bone formation-related factors (BMP-2, Runx2) significantly decreased (*P* < 0.05).

**Conclusions:**

Under the inflammatory environment induced by LPS, PI3K participates in the occurrence and development of chronic apical periodontitis by regulating the proliferation and differentiation of osteoclasts and osteoblasts.

## Background

Apical periodontitis (AP) is a prevalent infectious disease resulting from the interaction between microbial factors and host immune response [1–6]. Gram-negative bacteria predominate in root canals of teeth with periapical lesions, and among the virulence factors of gram-negative bacteria, lipopolysaccharides (LPS), or endotoxins, are the most potent stimuli for the release of inflammatory mediators by immune cells [7–12]. The release of inflammatory mediators and cytokines leads to inflammation and bone tissue destruction [13–16]. The dynamic balance of osteoclasts and osteoblasts plays an important role in the apical bone remodeling process [2, 17]. Based on differences in root-canal infection with different bacteria or virulence factors, the immunological response causing apical periodontitis will vary in acute or chronic inflammation [12]. By establishing an experimental animal model of apical periodontitis, the expression of related inflammatory factors or signaling pathways at different stages can be observed.

Phosphoinositide 3-kinase (PI3K) is located inside cells, and PI3Kγ deficiency reduces the number of osteoclasts in vivo and impairs the formation of osteoclasts in vitro [18], which leads to increased bone mass in mice. This indicated that PI3K may play an important role in promoting osteoclast formation. PI3K activates Akt and downstream proteins such as nuclear factor kappa-B (NF-κB) and glycogen synthase kinase3β (GSK3β), which are involved in regulating cell survival, proliferation, apoptosis and angiogenesis [19, 20]. Akt is a serine/threonine kinase, also known as protein kinase B (PKB), which is an important downstream target kinase in the PI3K signaling pathway [20]. Studies by Kawamura et al. [21] have shown that mice lacking Akt1 have reduced bone mass, and the lack of Akt1 in osteoclasts leads to autonomic cell dysfunction and impaired bone resorption. Ghosh-Choudhury et al. [22] showed that bone morphogenetic protein-2 (BMP-2) induces osteoblast differentiation by activating the PI3k/Akt pathway, but other studies showed that inhibitors of the PI3K pathway (Ly294002) not only fail to block the osteoblast phenotype induced by BMP-2, but also potentiate the effect of BMP-2 on late osteoblast markers, such as osteocalcin expression [23]. Lin et al. [24] showed that inhibiting the PI3K/AKT signaling axis by the PI3K inhibitor LY294002 prevents aberrant bone formation and attenuated articular cartilage degeneration.

So far, the results of research into the role of PI3K in bone resorption or bone formation are not completely consistent, and the role of PI3K in apical periodontitis caused by bacteria remains especially unclear. The hypothesis of this study was that PI3K may play a role during different stages of apical periodontitis progression, and that PI3K inhibitors inhibit formation of osteoclasts and promote differentiation of osteoblasts, thus promoting bone formation. Therefore, this study aimed to investigate the role of PI3K in apical periodontitis through in vivo and in vitro experiments.

## Methods

### Clinical sample collection and experiments

This study was conducted in full accordance with the World Medical Association Declaration of Helsinki and was approved by the Ethics Committee of the Affiliated Stomatological Hospital of Dalian Medical University School of Stomatology (Approval number: 2021005). Participants were patients who were seen by a doctor at the Department of Endodontics or Maxillofacial Surgery of the Dental Hospital of Dalian Medical University between January 2021 and December 2021 due to chronic apical periodontitis. The clinical cases were all apical periodontitis caused by caries-derived infection, and the diagnosis was mainly based on X-ray, with a periapical index (PAI) score ≥ 3 [25, 26]. A total of 26 periapical tissue samples were collected from patients who needed apical surgery or tooth extraction due to apical periodontitis. Among these patients, 14 were males and 12 were females, aged 22–53 years. At the same time, healthy periodontal ligament tissues from 10 patients after extraction of premolars or third molars as part of orthodontic treatment during the same period were collected as the control group, comprising six males and four females, aged 19–54. The subjects were fully informed and provided signed consent to provide tissue samples for research. The chronic apical periodontitis patients were diagnosed based on clinical manifestations and X-rays and they were in good health with no systemic diseases or immune system diseases. Exclusion criteria were: ① the tooth was affected by periodontal disease or combined periodontal and pulpal disease; ② antibiotics or non-steroidal anti-inflammatory drugs had been taken in the 3 months before tooth extraction; ③ allergies; ④ refusal to participate in this study. Each clinically-obtained tissue sample was placed into an Eppendorf (EP) tube containing 0.5 mL Trizol and stored at − 80 °C for real-time quantitative polymerase chain reaction (RT-qPCR).

### In vivo experiments

A total of 25 C57BL/6L female mice, 6–8-weeks old, were purchased from the Experimental Animal Center of Dalian Medical University. The animal experiments were approved by the Ethics Committee of Dalian Medical University (approval number: 2021006). An intraperitoneal injection of Ketamine (90–150 mg/kg) + Xylazine (7.5–16 mg/kg) was administered to anesthetize the mice, then apical periodontitis was established in the mandibular first molar by exposure of the pulp cavity [27]. The mice were fed on standard chow and allowed free access to food and water. At 0, 1, 2, 3, and 4 weeks after opening the pulp cavity, five mice were randomly selected for euthanasia. The mouse mandibular periapical tissue was prepared for hematoxylin–eosin (HE) staining, and the expression of relevant proteins was analyzed by immunohistochemical staining, while enzymatic histochemical staining was used for osteoclast detection. The methods of HE staining and immunohistochemical staining were as described in our previous study [16]. The PI3K polyclonal antibody (Bioworld, Nanjing, China) and AKT polyclonal antibody (Bioworld, Nanjing, China) were both used at a dilution of 1:100. Histochemical staining was carried out using a tartrate-resistant acid phosphatase (TRAP) staining kit (Sigma-Aldrich, Missouri, USA) according to the manufacturer’s instructions. The expression of PI3K and Akt, and the appearance of osteoclasts in mice with apical periodontitis was calculated using the average optical density value at 450 nm [28, 29].

### In vitro experiments

#### Cell induction and identification

The mouse macrophage cell line RAW264.7 (gifted by the Laboratory of Stomatology, Wuhan University) and the mouse osteoblastic cell line MC3T3-E1 (purchased from Wuhan Prosei Life Technology) with good growth from 3 to 4 generations were used as experimental cells. After cell digestion and passage, 2 × 10^6^ cells/well were inoculated into wells of a six-well plate, and cultured in high-glucose Dulbecco’s modified Eagle’s medium (DMEM) containing 10% fetal bovine serum (Gibco, California, USA), antibiotics and 2 mmol/L glutamine at 37 °C in a humidified atmosphere of 5% CO2. After RAW264.7 cell culture for 24 to 48 h, 0.1 μg/mL RANKL induction solution was added. After induction for 5 days, the cells were stained for TRAP according to the instructions provided with the staining kit. The level of Acp5 mRNA was also determined to further confirm that osteoclast induction was successful.

After MC3T3-E1 cells were cultured for 24 to 48 h, 10 nmol/L dexamethasone, 10 mmol/L sodium β-glycerophosphate sodium and 50 μg/mL ascorbic acid were added to each well. After 7 days, half the cultures were removed and used for ALP staining, while the remainder were induced for 21 days before use for Alizarin red staining. The level of ALP mRNA was also determined to further confirm successful induction of osteoblasts.

### Analysis of gene and protein expression under LPS-mediated inflammation

Induced osteoclasts or osteoblasts were chosen and inoculated at a density of 2 × 10^6^ cells/well into a six-well plate. After allowing the cells to adhere for 24 h, the cell culture medium was replaced and fresh medium plus 100 ng/ml LPS (Sigma-Aldrich, Missouri, USA) was added to the experimental group [30], while the control group received vehicle alone. After 24 h of exposure, the cells were harvested and the gene expression levels of PI3K, Acp5 and NFATc1, and the osteoblast-related factors BMP-2 and Runx2, were analyzed by RT-qPCR, while western blotting was used to analyze the protein expression levels of these factors.

### Detection of the effect of a PI3K inhibitor on the PI3K/Akt signaling pathway

Osteoclasts or osteoblasts of the third or fourth generation were seeded at 2 × 10^3^ cells/well into 96-well plates. After the cells were allowed to adhere, the original culture medium was discarded, and inhibition of PI3K activity with the specific inhibitor LY294002 (Cell Signaling Technology, Boston, USA) at 0 or 20 μmol/L, was added to the cells. After a further 24 h of culture, CCK-8 (APExBIO, Houston, USA) reaction solution was added, and cells were incubated at 37 °C in a 5% CO_2_ incubator for 1 h, then the OD value at 450 nm was measured using a microplate reader.

Osteoclasts or osteoblasts in good condition were selected to inoculate into a 6-well plate at a density of 2 × 10^6^ cells/well. After the cells were allowed to adhere for 24 h, the cell culture medium was replaced, and LY294002 was added at 0 or 20 μmol/L to the control or experimental groups, respectively. After a further 24 h, the cells were harvested for analysis. The effect of the PI3K inhibitor was analyzed by RT-qPCR to detect the gene expression of Akt, Acp5 and NFATc1 in osteoclasts, and Akt, BMP-2 and Runx2 in osteoblasts. Western blotting was used to analyze the protein expression of p-Akt, Akt, TRAP and NFATc1 in osteoclasts, p-Akt, Akt, BMP-2 and Runx2 in osteoblasts.

### Real-time quantitative polymerase chain reaction (RT-qPCR)

RNA was extracted from tissues or cells according to the instructions provided with the total RNA extraction kit (Takara, Shiga, Japan), and reverse transcribed to cDNA. Each cDNA sample was added to an eight-tube and centrifuged to mix. After mixing, the tube was placed in a qPCR reactor to carry out the PCR reaction. The RT-qPCR program consisted of a heating step at 95 °C for 30 s followed by 40 cycles of 95 °C for 5 s and 56 °C for 30 s. The primer sequences of human-related genes are shown in Table 1 and those of mouse-related genes in Table 2. The primer sequences were all designed and produced by Shanghai Bioengineering Co., Ltd. (Shanghai, China). The 2^−△△Ct^ method was used to calculate the relative expression of each target gene according to the Ct value of the internal control and target gene.

**Table 1:** Primer sequences of human-related genes

Primer	Sequences	Sequence ID:
GAPDH F	GCACCGTCAAGGCTGAGAAC	NM_001256799.3
GAPDH R	TGGTGAAGACGCCAGTGGA	
PI3K F	AGCATTGGGACCTCACATTACACA	NM_001256045.2
PI3K R	ACTGGAAACACAGTCCATGCACATA	
TRAP F	CCTACCCACTGCCTGGTCAA	NM_001111034.3
TRAP R	CCTACCCACTGCCTGGTCAA	
NFATc1 F	GAAGACCGTGTCCACCACCA	NM_001278669.2
NFATc1 R	CGAAGTTCAATGTCGGAGTTTCTG

**Table 2:** Primers sequence of mouse related genes

Primer	Sequences	Sequence ID:
GAPDH F	AAATGGTGAAGGTCGGTGTG	NM_001289726.1
GAPDH R	TGAAGGGGTCGTTGATGG	
PI3K F	CCCATGGGACAACATTCCAA	NM_001024955.2
PI3K R	CATGGCGACAAGCTCGGTA	
TRAP F	GGGTCACTGCCTACCTGTGT	NM_001102404.1
TRAP R	TCATTTCTTTGGGGCTTATCTC	
ALP F	GGTCTGTGTTCGTAAGGGTGA	NM_001287172.1
ALP R	AGGTCAAGAATAAGGTGTAGTC	
NFATc1 F	TCAGTTCCCGTCCGTTCCTA	NM_001164109.1
NFATc1 R	TTCAGAGTGGTGTCCCGAGT	
BMP2 F	CTCCAACGGTGCTAGGTCAGT	NM_007553.3
BMP2 R	ATTGGTACGGTATCACGTCTGAGAC	
Runx2 F	GGAGACAACATTTATGACGAACGTC	NM_001145920.2
Runx2 R	TTCTACTCGCTGCACTCGG	

### Western blotting

Total protein was extracted from experimental samples of clinical tissues or harvested cells. The protein concentration and protein denaturation were determined using a BCA assay kit (Sigma-Aldrich, Missouri, USA), according to the instructions supplied. Sodium dodecyl sulfate–polyacrylamide gel electrophoresis (SDS-PAGE) was performed according to the electrophoresis gel preparation kit, then the separated proteins were transferred to polyvinylidene difluoride membranes, the blots were cut prior to hybridisation to the antibodies, and incubated with the primary antibody at 4 °C overnight. The antibodies used were as follows: rabbit monoclonal anti-GAPDH (1:1000 dilution; Bioworld, Nanjing, China), rabbit monoclonal anti-PI3K (1:500 dilution; Bioworld, Nanjing, China), rabbit monoclonal anti-Akt (1:5000 dilution; Cell Signaling Technology, Boston, USA), rabbit monoclonal anti-p-Akt (1:5000 dilution; Abcam, Cambridge, UK), rabbit monoclonal anti-TRAP (1:500 dilution; ABclonal, Wuhan, China), rabbit monoclonal anti-NFATc1 (1:500 dilution; Elabscience Biotechnology, Wuhan, China), rabbit monoclonal anti-BMP-2 (1:500 dilution; Bioworld, Nanjing, China), rabbit monoclonal anti-Runx2 (1:5000 dilution; Bioworld, Nanjing, China). After washing three times with TBST on a shaker, each time for 10 min, the membranes were incubated with the secondary antibody (1:1000 dilution; Bioworld, Nanjing, China). After further washes with TBST, membranes were incubated with ECL luminescent solution (Thermo Fisher Scientific, Waltham, USA), and a Bio-Rad gel imaging system was used to capture the western blot results. Image Lab software (Bio-Rad) was then used to analyze the results. After the protein bands were obtained in the experiment, the gray value of each band was obtained using Image Lab software. The internal reference protein GAPDH was used for normalization, and the ratio of the value of the target protein to GAPDH was the relative gray value of that protein. Each experiment was repeated three times, and the obtained relative gray values were used for statistical analysis.

### Statistical analysis

In vivo experiments, HE staining, immunohistochemical staining and TRAP staining have 0, 1, 2, 3, 4 time points, 5 animals at each time-point, 5 non-consecutive sections per animal, a total of 25 data-points in each group for statistical analysis. For in vitro experiments, each experiment was undertaken at least three times unless stated otherwise. SPSS 17.0 software was used to analyze the data. Measurement data were expressed as mean ± SD, comparisons between two groups were performed using Tukey's test (α = 0.05), while comparisons between multiple groups were performed using one-way analysis of variance. The significance level for all analyses was set at 5% (α = 0.05). Bivariate correlation analysis of the relationship between PI3K, AKT and osteoclasts was performed.

## Results

### Expression of PI3K, Acp5 and NFATc1 in clinical samples

**Fig. 1:**
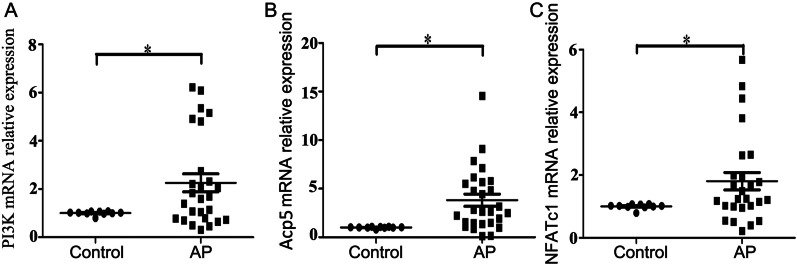
Gene expression of PI3K (**A**), Acp5 (**B**) and NFATc1 (**C**) in clinical samples of chronic apical periodontitis. *P < 0.05 represents a significant difference between the groups of apical periodontitis and healthy tissues

As shown in Fig. 1, the expression of PI3K (*P *= 0.009, Fig. 1A), Acp5 (*P *< 0.001, Fig. 1B) and NFATc1 (*P *= 0.003, Fig. 1C) genes were all higher in the chronic apical periodontitis samples than in healthy periodontal ligament tissue, and the difference was statistically significant.

### Expression of PI3K and Akt in a mouse model of apical periodontitis

**Fig. 2:**
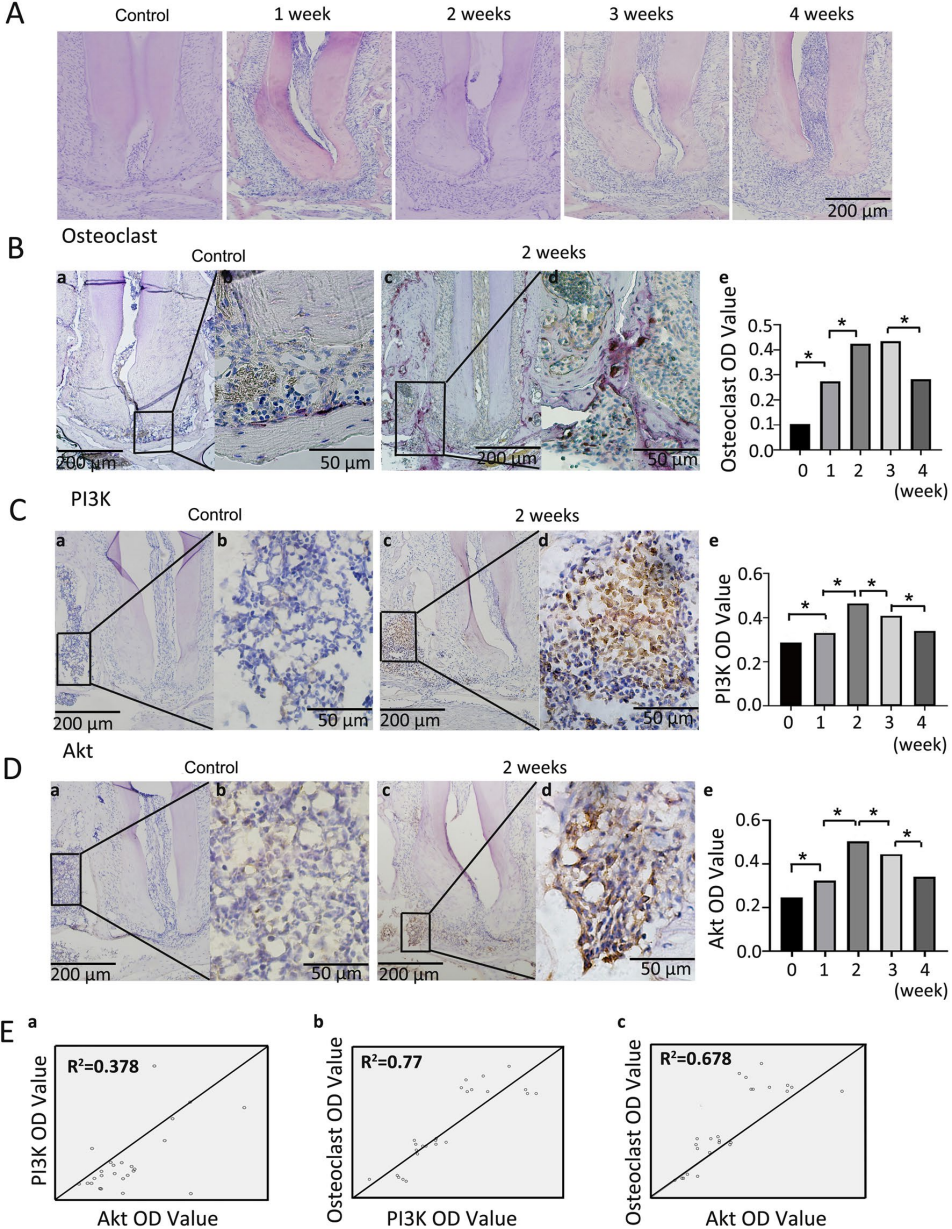
Expression of PI3K and Akt in a mouse model of apical periodontitis. **A** HE staining of the mouse apical tissues of the mandibular first molars at 1, 2, 3, and 4 weeks after pulp opening and normal control group (×100). **B** a, b, c and d, TRAP staining of the mouse periapical tissues in the control group and 2nd week after pulp opening, the positive cells were distincitve by very large cellular sizes(≥ 3 multiple nuclei), wine red; e, statistical analysis of the OD values of positive osteoclasts in each experimental group, * represent significant differences between groups (*P* < 0.05). **C** a, b, c and d, expression of PI3K in mouse periapical tissues in the control group and 2nd week after pulp opening, the positive cells are tawny; e, statistical analysis of the OD values of PI3K in each experimental group, * represent significant differences between groups (*P* < 0.05). **D** a, b, c and d, expression of Akt in the mouse periapical tissues in the control group and 2nd week after pulp opening, the positive cells are tawny; e, statistical analysis of the OD values of Akt in each experimental group, * represent significant differences between groups (*P* < 0.05). **E** Correlation analysis between PI3K OD value, AKT OD value and osteoclast OD value. HE, 10× original magnification. Immunohistochemical,10× or 40× original magnification

The results of HE staining and TRAP staining are shown in Fig. 2A, B. With the continuous progression of periapical inflammation, the exudation of periapical tissue and the extent of bone destruction gradually increased. Immunohistochemical staining (Fig. 2C, D) showed that cells positive for PI3K and Akt were yellow–brown, and their expression levels were highly consistent. Correlation analysis (Fig. 2E) showed that there was a high correlation between PI3K OD value and osteoclast OD value (r = 0.883, *P* < 0.001), and there was also a high correlation between AKT OD value and osteoclast OD value (r = 0.827, *P* < 0.001), while there was a moderate correlation between PI3K OD value and AKT OD value (r = 0.615, *P* < 0.001).

### Cell induction and identification

**Fig. 3:**
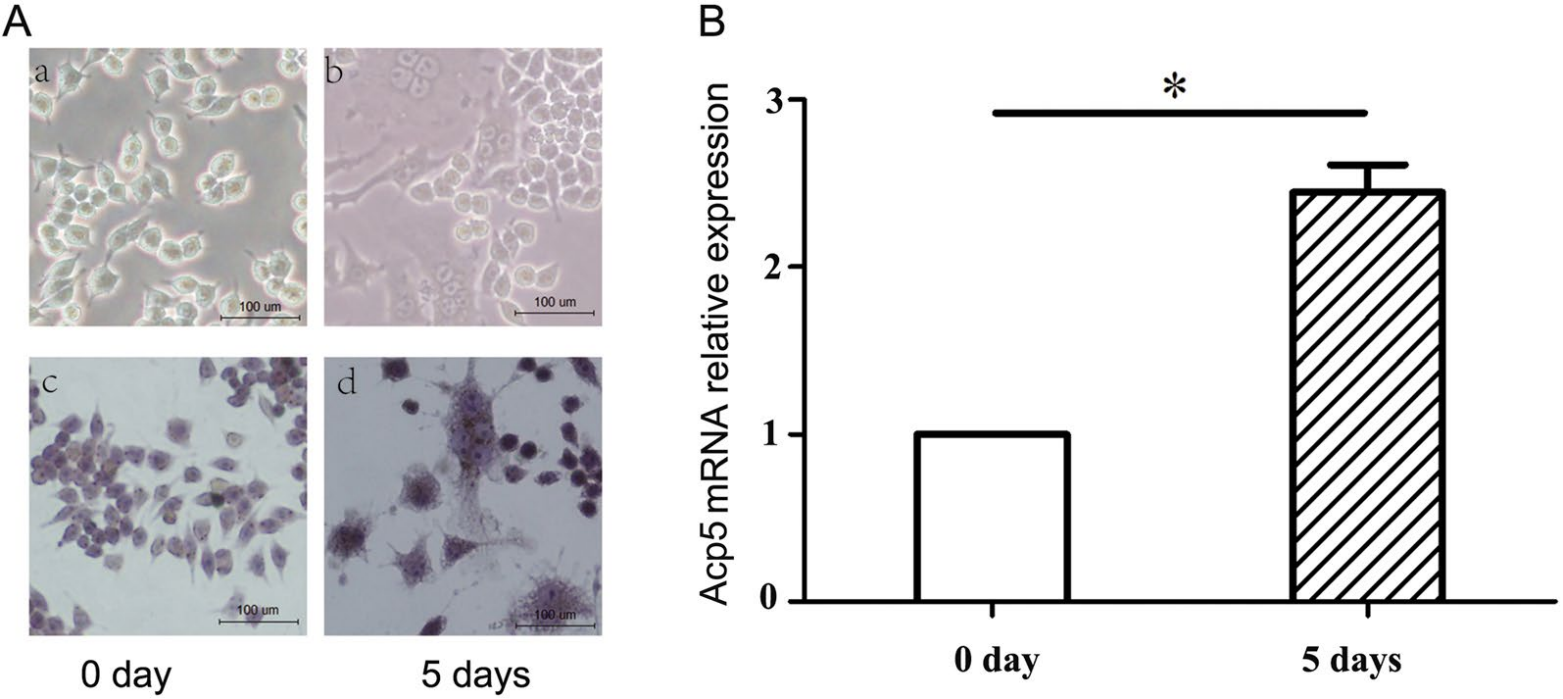
Osteoclast induction and identification. **A** a and b, RAW264.7 cells after 0 and 5 days of induction, viewed under a light microscope (×200); c and d, TRAP staining of RAW264.7 cells at 0 and 5 days of induction (×200); **B** Acp5 mRNA expression of RAW264.7 cells at 0 and 5 days of induction.*P = 0.0009. 40× original magnification

As shown in Fig. 3, after 5 days of induction of RAW264.7 cells, TRAP staining stained the cytoplasm wine-red with acid phosphatase granules, and most of the cells were giant cells with three or more nuclei. RT-qPCR analysis revealed that the mRNA expression of Acp5 was significantly higher after 5 days of induction of RAW264.7 cells compared with 0 days (*P* < 0.05). The above results indicate that RAW264.7 cells were successfully induced to form osteoclasts.

**Fig. 4:**
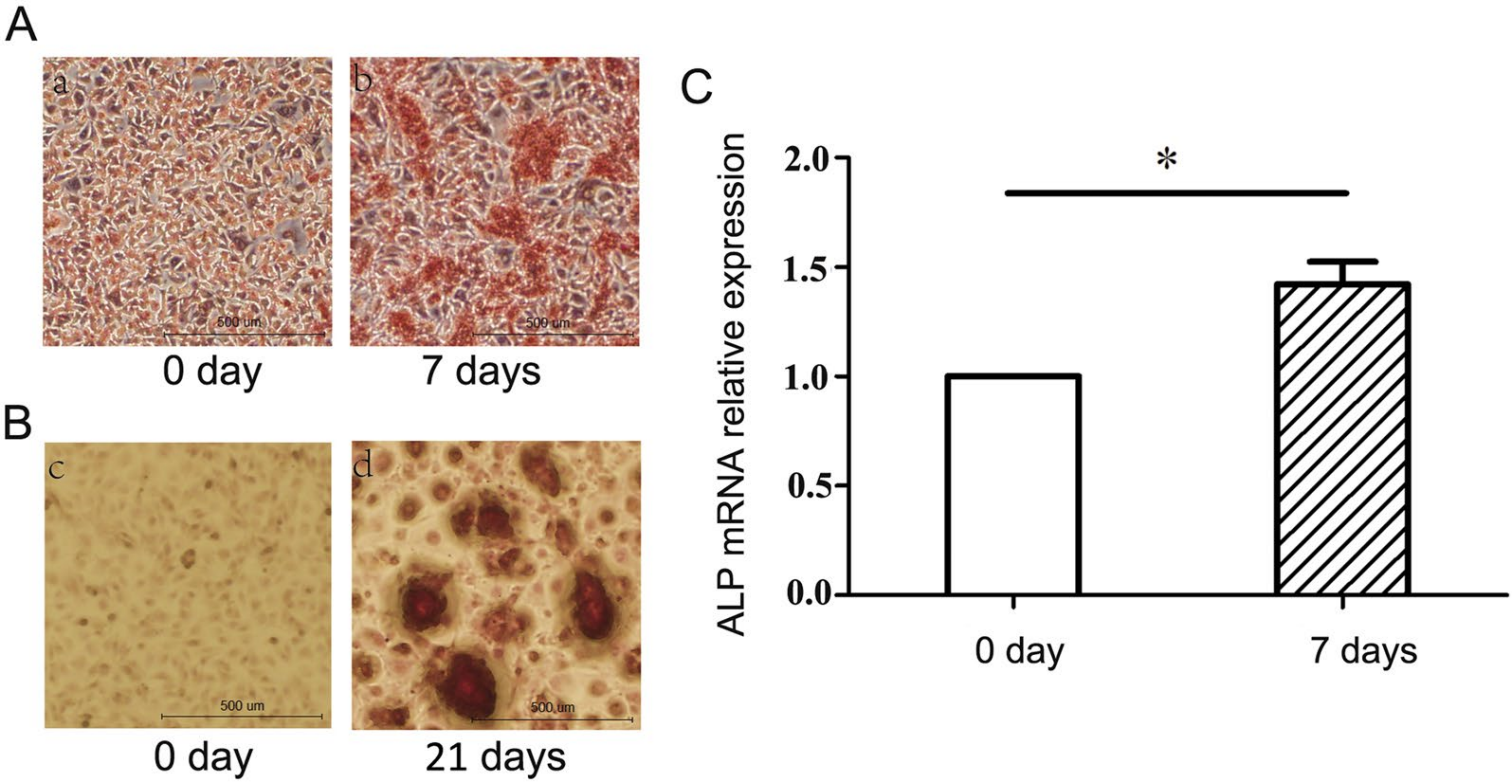
Osteoblast induction and identification. **A** a and b, ALP staining of MC3T3-E1 cells after 0 and 7 days of induction, viewed under a light microscope (×40); c and d, Alizarin red staining of MC3T3-E1 cells at 0 and 21 days of induction, viewed under a light microscope (×40); **B** the ALP mRNA expression results of MC3T3-E1 cells at 0 and 7 days of induction. **C** Statistical analysis of ALP mRNA expression in osteoblasts. *P = 0.015

The results of ALP staining are shown in Fig. 4. Compared with 0-day MC3T3-E1 cells, MC3T3-E1 cells induced for 21 days contained a large number of flaky, dark red calcium nodules, and the mRNA expression of ALP was significantly increased after induction of MC3T3-E1 cells for 7 days compared with day 0 (*P* < 0.05). The above results indicate that MC3T3-E1 cells were successfully induced to form osteoblasts.

### Expression of PI3K and related factors in cells under LPS-mediated inflammation

**Fig. 5:**
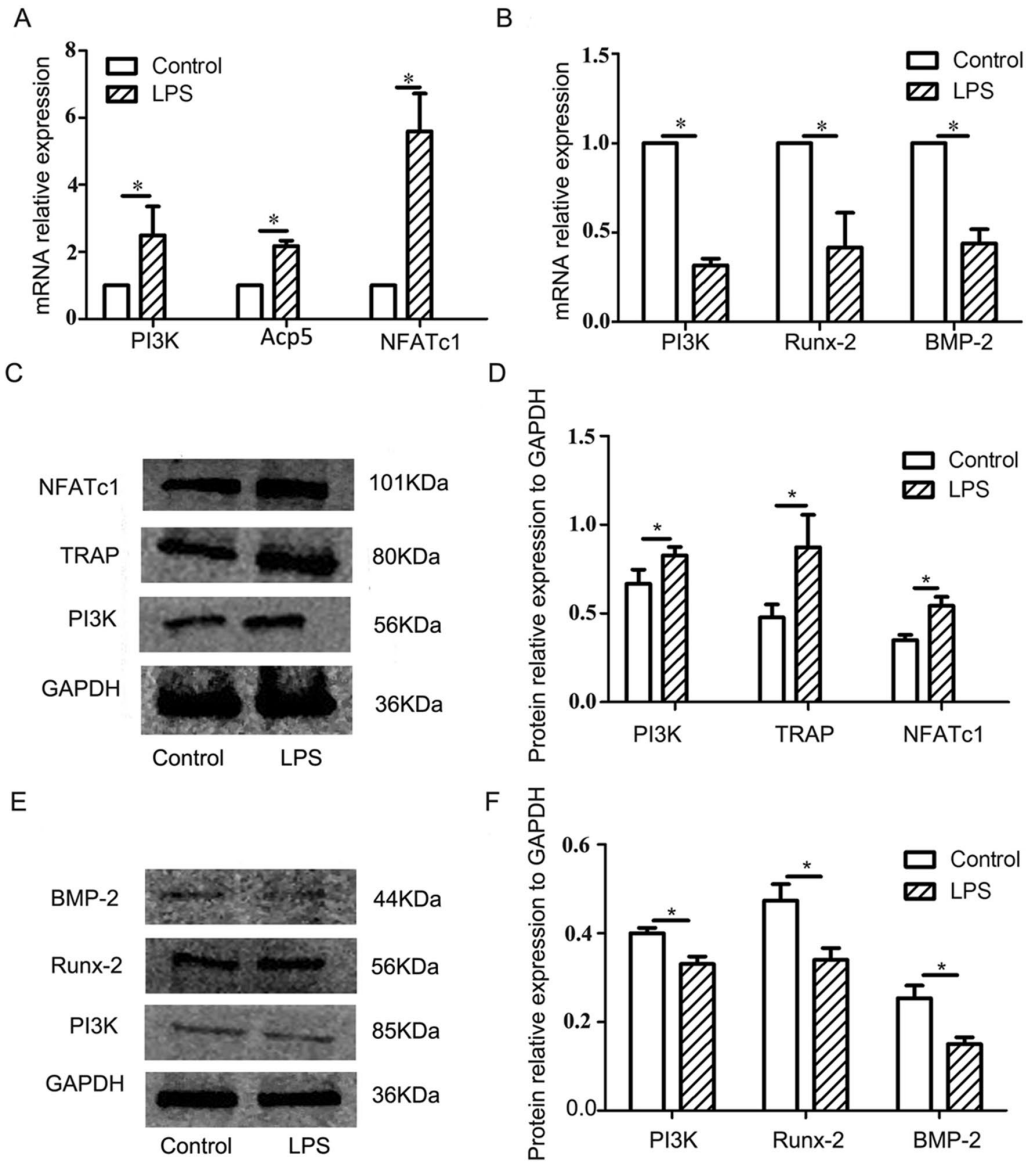
Detection of related genes and proteins after treatment of osteoclasts and osteoblasts with LPS. **A** mRNA expression of PI3K (*P* = 0.038), Acp5 (*P* < 0.001) and NFATc1 (*P* = 0.002) in osteoblasts under the action of LPS; **B** mRNA expression of PI3K (*P* < 0.001), BMP-2 (*P* = 0.002) and Runx2 (*P* = 0.039) in osteoblasts under the action of LPS. **C** Expression of PI3K, TRAP and NFATc1 proteins in osteoclasts under the action of LPS; **D** statistical analysis of PI3K (*P* = 0.032), TRAP (*P* = 0.022) and NFATc1 (*P* = 0.028) protein expression in osteoclasts; **E** expression of PI3K, BMP-2 and Runx2proteins in osteoblasts under the action of LPS; **F** statistical analysis of PI3K (*P* = 0.045), Runx2 (*P* = 0.018) and BMP-2 (*P* = 0.021) protein expression in osteoblasts.*P < 0.05, representing a significant difference between the different groups

Gene and protein expression results of cells cultured in an LPS-mediated inflammatory microenvironment are shown in Fig. 5. In the experimental group of osteoclasts, the mRNA levels of PI3K and osteoclast-related factors Acp5 and NFATc1 were significantly increased (*P* < 0.05) (Fig. 5A), while the protein levels of PI3K, TRAP and NFATc1 (Fig. 5C, D) were consistent with the RT-qPCR results (*P* < 0.05).

In osteoblasts, gene expression analysis (Fig. 5B) showed that mRNA levels of PI3K and the osteogenic factors BMP-2 and Runx2 were significantly reduced (*P* < 0.05). The protein levels of PI3K, BMP-2 and Runx2, analyzed by western blotting, were consistent with RT-qPCR results (Fig. 5E, F).

### The effect of PI3K inhibitors on cell proliferation, differentiation and the PI3K/Akt signaling pathway

**Fig. 6:**
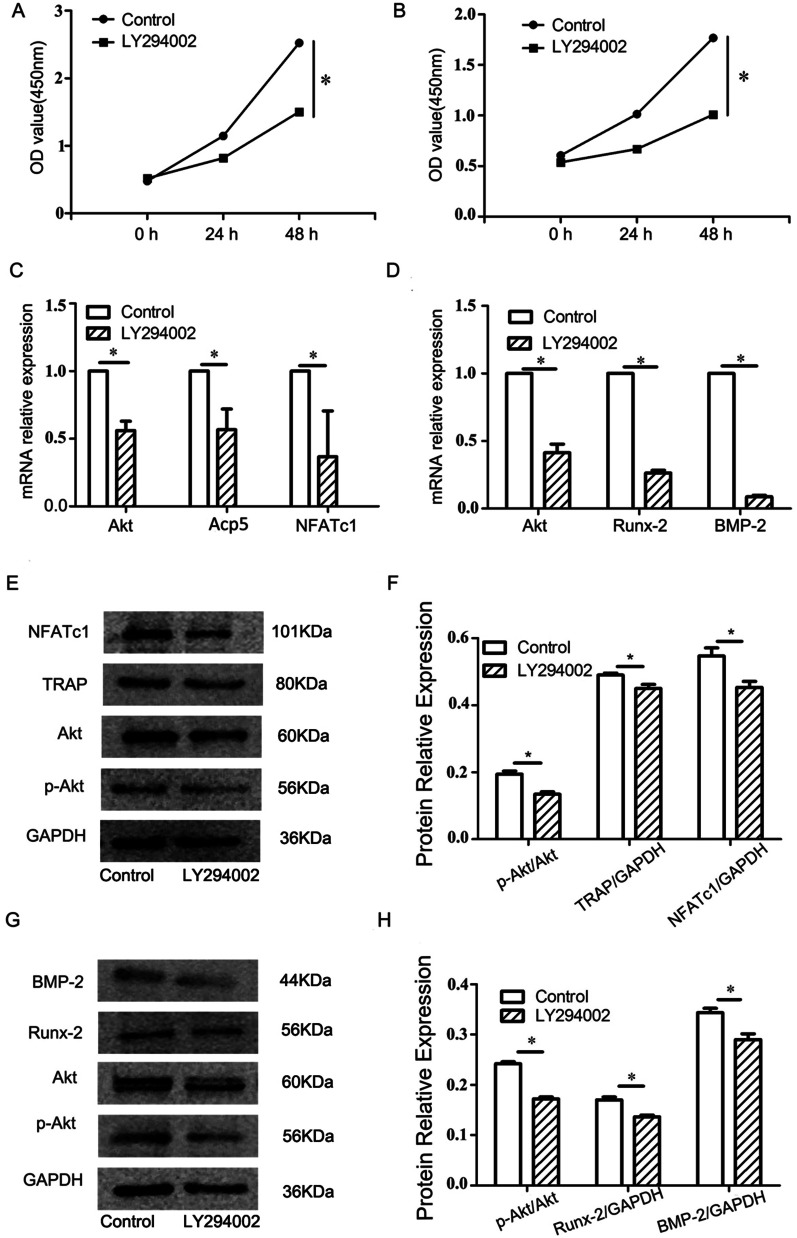
Effects of LY294002 on the proliferation activity and related gene and protein expression of osteoclasts and osteoblasts. **A** The cell proliferation of osteoclasts treated with LY294002; **B** the cell proliferation of osteoblasts treated with LY294002; **C** statistical analysis of mRNA expression of Akt (*P* = 0.023), Acp5 (*P* = 0.019) and NFATc1 (*P* = 0.011) in osteoblasts treated with LY294002; **D** statistical analysis of mRNA expression of Akt (*P* = 0.019), BMP-2 (*P* = 0.012) and Runx2 (*P* = 0.003) in osteoblasts treated with LY294002. **E** The expression of PI3K, TRAP and NFATc1 proteins in osteoclasts treated with LY294002; **F** statistical analysis of p-Akt (*P* = 0.009), TRAP (*P* = 0.039) and NFATc1 (*P* = 0.032) proteins expressed in osteoclasts; **G** the expression of PI3K, BMP-2 and Runx2 proteins in osteoblasts treated with LY294002; **H** statistical analysis of p-Akt (*P* = 0.027), Runx2 (*P* = 0.031) andBMP-2 (*P* = 0.028) proteins expressed in osteoblasts. *P < 0.05 represents a significant difference between the control and experimental groups

The results of CCK-8 assay are shown in Fig. 6A, B. The RT-qPCR results are shown in Fig. 6C, D, The mRNA levels of Akt and the osteoclast differentiation markers Acp5 and NFATc1 decreased significantly in the osteoclast experimental group (*P* < 0.05), while mRNA levels of Akt, BMP-2 and Runx2 were similarly reduced in the osteoblast experimental group (*P* < 0.05). The ratio of p-Akt/Akt protein in the osteoclast experimental group decreased, and the protein expression levels of the osteoclast differentiation markers TRAP and NFATc1 corresponded to the RT-qPCR results, with the expressions of all being significantly decreased (*P* < 0.05) (Fig. 6E, F). As shown in Fig. 6G, H, the ratio of p-Akt/Akt protein in the osteoblast experimental group decreased and the protein levels of the bone formation-related factors BMP-2 and Runx2 also all decreased significantly, consistent with the results of RT-qPCR (*P* < 0.05).

## Discussion

In this study, high expression of PI3K was found in both human apical periodontitis samples and periapical tissues of mouse apical periodontitis. Through the application of PI3K inhibitors, it was further verified that PI3K promotes the formation of osteoclasts, however, PI3K inhibitors also reduced the differentiation and maturation of osteoblasts. Thus, we reject the initial hypothesis that PI3K plays an important role in bone formation. PI3K plays a role in promoting cell differentiation and maturation in both osteogenic precursor cells and osteoclast precursor cells.

Our results showed that PI3K was consistent with Acp 5 and NFATc1 in that they were all highly expressed in human chronic apical periodontitis tissues. In the clinical samples collected in this study, the periapical index scores ≥ 3, which were refractory apical periodontitis requiring apical surgery or tooth extraction due to apical periodontitis, indicating that PI3K may play an important role in the bone destruction induced by severe inflammation.

An induced animal model of periapical periodontitis allows better study of the role of PI3K in bone homeostasis. An animal model of apical periodontitis can be established by methods including root canal exposure to the oral cavity, or inoculation of bacteria [31, 32], LPS [33, 34] or lipopolysaccharide (LTA) [16] into the pulp cavity or root canal [35, 36]. The pathological results of apical periodontitis induced by different methods are not the same; apical periodontitis induced by root canal exposure to the oral cavity is characterized by the recruitment of inflammatory cells and bone tissue resorption, whereas inoculation of LPS induces inflammatory cell recruitment and less bone loss [33]. The present study focused on the role of PI3K in the bone destruction of apical periodontitis, so the cavity exposure method was used to establish the mouse model of apical periodontitis.

Micro-computed tomography (micro-CT) is the method normally used to determine the periapical bone loss in induced apical periodontitis. HE staining is the main method to detect the expression of periapical inflammatory cells, but it cannot accurately determine the volume of periapical bone loss. The bone loss observed by micro-CT is three-dimensional, while that observed by HE staining is two-dimensional and only reflects the area. A previous study carried out by our group used micro-CT to compare the volume of apical periodontitis-induced bone loss in cathepsin C (Cat C) knock-down (KD) mice and normal mice [15], but the focus of the present study was not to detect bone loss. Therefore, only HE staining was used to verify the successful establishment of the animal model of apical periodontitis.

In recent years, studies on the PI3K/Akt signaling pathway have found that this pathway is involved in the occurrence and development of pathological bone diseases such as osteoporosis, osteoarthritis, and osteosarcoma [37–39]. The present study also showed that PI3K and Akt expression was significantly increased during apical periodontitis progression, especially in the active phase of bone resorption, when the expression of PI3K and Akt peaked. Studies have shown that the animal model of apical periodontitis established with cavity exposure to the oral environment is initially characterized by the thickening of the apical periodontal ligament in the first 7 days, the recruitment of neutrophils and macrophages at 14 days, the presence of bone resorption at 21 days, and extensive periapical bone resorption at 28 days [33]. Our results showed that osteoclast numbers peaked when inflammation progressed to week 2, with no statistically-significant difference between week 3 and week 2. There were some differences during the earliest time of bone destruction, but whether it is related to the sex of the selected mice needs further study.

The inflammatory environment induces the proliferation and differentiation of osteoclast precursor cells by several mechanisms, leading to bone resorption in apical periodontitis. To study the role of PI3K in bone resorption, osteoclasts and osteoblasts were treated with LPS to establish an inflammatory microenvironment. The results showed that the expression of PI3K and osteoclast-related factors in osteoclasts was significantly increased, while in osteoblasts, the expression of PI3K and osteoblast-related factors was significantly decreased, and the expression trends of proteins and genes were exactly the same, since protein is the result of gene expression, and gene expression is also regulated by the corresponding protein activator or repressor. The trend of the two was consistent in this study, which further indicated that in an inflammatory microenvironment, the main role of PI3K is to promote bone resorption and inhibit bone formation. The effects of PI3K on osteoblasts are not consistent between inflammatory conditions and normal conditions, since under normal conditions, bone morphogenetic protein-2 (BMP-2) induced osteoblast differentiation by activating the PI3k/Akt pathway [22].

To further study the mechanism of PI3K in bone destruction, we used the PI3K inhibitor LY294002 to investigate its effect on osteoclasts. LY294002 is an ATP-competitive inhibitor specific to PI3K that blocks downstream signaling pathways [40, 41]. Our results showed that LY294002 inhibited the proliferation and differentiation of osteoclasts, indicating that PI3K participates in the maturation of osteoclasts. These findings were similar to the results of Lee et al. [42]. At the same time, LY294002 was applied to osteoblasts, and the results showed that the differentiation of osteoblasts and the expression of osteogenic marker factors were reduced. These results are similar to those of Ghosh-Choudhury N et al. [22] and Fujita et al. [43]. However, the results of Viñals et al. [23] reported that PI3K has a negative role in osteoblast differentiation. The main reason may be the difference between cells and inducers. We used the mouse osteoblastic cell line MC3T3-E1, and the cell differentiation induction solution included dexamethasone, glycerophosphate sodium and ascorbic acid. Viñals et al. [23] used C2C12 mouse cells with or without BMP-2 induction.

Although the study reported in this paper confirmed that the PI3K/AKT signaling pathway is highly active in apical periodontitis, PI3K promotes the proliferation of osteoclasts and inhibits the differentiation and maturation of osteoblasts in the inflammatory environment. However, there are still some issues that have not been resolved, and further research is needed. For example, whether PI3K inhibitors can be used to treat apical periodontitis in mice, and what effect the overexpression of PI3K has on the differentiation and maturation of osteoclasts and osteoblasts, are questions that remain to be addressed in future work.

## Conclusions

Our study confirmed that PI3K inhibitors can reduce the expression of osteoblasts and osteoclast markers, indicating that PI3K is a key factor in promoting bone homeostasis under normal conditions. In the inflammatory state, PI3K is highly expressed in osteoclasts, especially in apical periodontitis. These lines of evidence suggest a crucial role of the PI3K/AKT signaling pathway in promoting bone resorption in the inflammatory state. Further studies on blocking the PI3K signaling pathway may determine an effective way to treat apical periodontitis bone destruction.

## Data Availability

All the data generated during the study are available in the manuscript itself.
